# COlour vision assessment for the Detection of Early diabetic Retinopathy (CODER): A study protocol

**DOI:** 10.1371/journal.pone.0359653

**Published:** 2026-10-09

**Authors:** Vaishaali Gunalan, Vinitha Mingi, Brijesh Takkar, Padmaja Kumari Rani, Amithavikram R. Hathibelagal

**Affiliations:** 1 Brien Holden Institute of Optometry and Vision Sciences, L V Prasad Eye Institute, Hyderabad, Telangana, India; 2 Anant Bajaj Retina Institute, L V Prasad Eye Institute, Hyderabad, Telangana, India; 3 Prof. Brien Holden Eye Research Centre, L V Prasad Eye Institute, Hyderabad, Telangana, India; PLOS: Public Library of Science, UNITED KINGDOM OF GREAT BRITAIN AND NORTHERN IRELAND

## Abstract

**Introduction:**

Diabetic retinopathy (DR) remains one of the leading causes of preventable vision loss. However, high-contrast visual acuity, which is routinely used in the clinic, is often preserved during the early stages of the disease, limiting its usefulness in detecting early functional deficits. Colour vision is a sensitive marker for detecting these early retinal changes. This study aims to investigate whether the Colour Assessment and Diagnosis (CAD) test, a quantitative measure of chromatic sensitivity, can serve as a functional biomarker for the early detection of DR and can reflect the severity of the disease.

**Methods and analysis:**

This prospective observational study will be conducted at the L V Prasad Eye Institute, Hyderabad, India. Adults aged 18 years and above will be recruited into three groups: healthy controls, individuals with diabetes without diabetic retinopathy, and individuals with diabetic retinopathy. All participants will undergo a comprehensive ophthalmic examination, colour vision testing, fundus photography, optical coherence tomography (OCT), optical coherence tomography angiography (OCTA), and systemic metabolic investigations, including glycaemic, renal, and lipid profiles. The primary outcome measures are the Red–Green (RG) and Yellow–Blue (YB) Colour thresholds. Secondary outcome measures include retinal structural parameters and vascular parameters, such as retinal thickness, vessel density, and perfusion density. Systemic metabolic parameters, including HbA1c, random blood glucose, renal function, and lipid profile, will be evaluated as independent variables. Statistical analyses will include correlation, multiple regression, and receiver operating characteristic (ROC) curve analyses to evaluate the diagnostic accuracy of the CAD test in the detection of early DR.

**Conclusion:**

The current study is expected to provide evidence whether quantitative colour vision assessment can act as a functional marker of early retinal changes in diabetes and diabetic retinopathy.

## Introduction

Type 2 diabetes is a rapidly increasing global health burden and remains a major public health concern. Globally, India ranks second, with 89.8 million individuals with diabetes and expected to grow up to 156.7 million by the year 2050 [1]. Among individuals with diabetes, the prevalence of DR is 17.4%. DR is a sight-threatening complication associated with prolonged hyperglycaemia [2]. In the working-age population (24–65 years), DR contributes substantially to preventable visual impairment and can have considerable economic consequences at both individual and societal levels.

Studies indicate that managing diabetes-related complications can consume up to 10% of an individual’s annual income, with the cost of treating severe DR being nearly three times higher than managing mild DR [3,4] As the incidence of DR rises, the economic burden on the healthcare system is expected to grow substantially. However, diagnosing the disease at an early stage can reduce the risk of blindness by 57% [5], with an appropriate intervention, making it critical to identify high-risk individuals before irreversible damage occurs. This highlights the urgent need to identify high-risk individuals with diabetes and detect diabetic retinopathy at an early stage, thereby enabling timely intervention and reducing the risk of visual impairment and blindness.

Currently, visual acuity (VA) and fundus evaluations are the most commonly used clinical tests for assessing treatment outcomes and monitoring disease progression. However, the routinely used high-contrast VA remains largely unaffected in the early stages of DR. At the same time, fundus changes progress slowly in the background, making VA an inadequate functional marker for early detection [6]. It is crucial to establish and validate sensitive and adoptable functional tests that can detect early disease changes and accurately reflect DR severity. Such tests could also serve as functional endpoints in clinical trials evaluating emerging treatment approaches.

Previous literature suggests that colour vision deficits may be present before the clinical manifestation of early DR-related changes [7–12].While colour vision deficits in diabetes have been widely reported, the existing evidence is limited by colour vision testing with varying protocols [13], varied study populations comprising both Type 1 and Type 2 diabetes [14], a predominant focus on Type 1 diabetes [15,16] and limited sample size [17]. Consequently, uncertainty remains regarding the nature and extent of colour vision impairment in individuals with Type 2 diabetes. Thus, the study protocol aims to employ the Colour Assessment and Diagnosis (CAD) test, a validated quantitative colour vision test [18] for assessing the severity and type of colour vision loss. Unlike conventional colour vision tests, the CAD test provides quantitative colour discrimination thresholds for yellow–blue (YB) and red–green (RG) by measuring true chromatic sensitivity independent of luminance cues [19]. The CAD test has a built-in normative database that allows comparisons with age-matched normative values [20]. It can be used to assess disease severity and as an outcome measure in various retinal diseases. [17,21]

The occurrence and progression of Diabetic Retinopathy depend strongly on several factors, including blood glucose control, typically characterised by HbA1c [22,23]; lipid profile [24]; and duration of diabetes [23]. Among these, HbA1c is one of the most important predictors of diabetic retinopathy, with a 10% reduction in HbA1c associated with a 43–45% lower risk of DR progression [25]. Although the CAD test has been validated as a quantitative measure of colour vision loss, it remains unclear whether these deficits in diabetes reflect systemic metabolic dysfunction or occur independently of it. Elucidating the relationship between CAD-derived RG and YB colour vision thresholds and systemic metabolic markers, such as glycosylated haemoglobin (HbA1c), may improve our understanding of how early the functional visual changes interact with systemic factors such as glycosylated haemoglobin (HbA1c). Furthermore, the diagnostic accuracy of the CAD test in detecting the earliest stages of diabetic retinopathy remains unknown, and whether the magnitude of colour vision deficits varies across different stages of DR severity. To address these knowledge gaps, we propose to evaluate i) how colour vision thresholds correlate with systemic measures and ii) the accuracy of the CAD test in identifying early DR and its association with different levels of disease severity. Understanding this link could play a pivotal role in understanding disease pathogenesis, its correlation with disease severity, and the role of systemic factors in relation to DR.

### Study aims and hypotheses

The current study aims to investigate whether the CAD test can serve as a biomarker for early DR detection and severity assessment of diabetic retinopathy.

The following are the hypotheses of the study:

- CAD test scores will correlate with glycaemic exposure (HbA1c) in patients with type 2 diabetes (Fig 1A).
- CAD will demonstrate high sensitivity and specificity in detecting early DR, compared to fundus photography (Fig 1B).
- CAD test scores will demonstrate stronger correlations with structural retinal changes (e.g., retinal thickness) than visual acuity, reflecting early functional impairment (Fig 1C).

**Fig 1:**
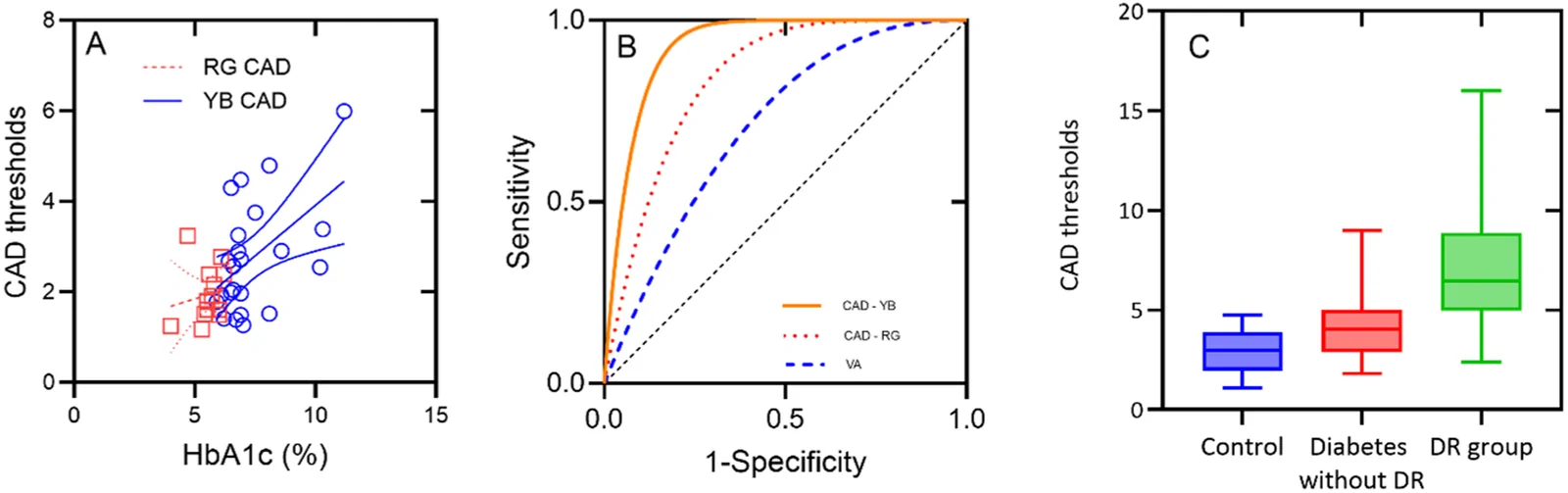
Study hypotheses for each of the study aims. (A) Relationship between glycaemic control (HbA1c, %) and chromatic detection thresholds along the red–green (RG, red) and yellow–blue (YB, blue) axes. Thresholds are expressed in CAD units. The prediction is that YB (Tritan) thresholds would show a stronger correlation with HbA1C, consistent with literature. (B) Receiver operating characteristic (ROC) curves showing the diagnostic utility of the RG and YB CAD test, and visual acuity for detecting early diabetic retinopathy. (C) CAD thresholds stratified by disease severity: healthy controls (Normal), diabetes without retinopathy (DM without DR), and diabetes with retinopathy (DM with DR). Box plots show the median (central line), interquartile range (box), and range (whiskers); It is hypothesized that CAD thresholds will progressively worsen with disease severity.

### Study objectives

The objectives of the study are 1) to evaluate the relationship between CAD test scores and glycaemic exposure (HbA1c), 2) to determine the sensitivity and specificity of CAD in detecting early DR, compared to standard fundus photography, and 3) to evaluate the structure-function relationship by determining whether CAD scores correlate with the severity of the disease (Table 1).

**Table 1:** Overview of study objectives, sample size, investigations, analyses, and expected outcomes for evaluating CAD scores in diabetic retinopathy screening and severity assessment.

Parameter	Objective 1	Objective 2	Objective 3
**Objective**	Evaluate the relationship between CAD test scores and glycemic exposure (HbA1c)	Determine the sensitivity and specificity of CAD in detecting early DR, comparing fundus photography	To test whether CAD scores will correlate with the severity of the disease
**Tests done**	HbA1c and random blood glucose tests will be measured.	Fundus photography	Optical Coherence Tomography (OCT), OCT Angiography (OCTA)
**Sample size**	Cases (diabetes no DR): 100; Controls: 100	Mild NPDR: 50; Controls (same as in objective 1): 100	Mild and moderate NPDR: 100; Severe NPDR: 50; Proliferative Diabetic Retinopathy (PDR): 50
**Analysis**	Correlation between CAD scores and HbA1c levels	Calculate sensitivity, specificity, predictive values and AUC of CAD for early detection of early DR	Assess structural, functional and vascular parameter correlations
**Expected outcomes**	Strength and direction of CAD scores and influence of glycemic exposure on colour vision	Determine the diagnostic accuracy of the CAD score in detecting early DR	Strength of correlation of CAD score with clinical DR severity

## Materials and methods

### Study design and participants

This will be a prospective, cross-sectional study. Participants will be adults aged 18 years and above. They will be categorized into three groups: A) controls, B) diabetes without DR, and C) diabetes with DR.

### Study setting

The study is being conducted at the L V Prasad Eye Institute, Kallam Anji Reddy Campus, Hyderabad. All the participants will undergo a comprehensive eye examination, including history documentation, visual acuity assessment, refraction, intraocular pressure measurement using Goldmann applanation tonometry, and dilated fundus examination using an indirect ophthalmoscope by an experienced retina specialist to rule out other retinal pathologies.

### Selection of participants

The participants aged 18 years and above are being screened and recruited based on the following inclusion criteria:

- Controls: Participants were selected if they self-reported normal systemic health. Best Corrected Visual Acuity (BCVA) of 20/30 or better in at least one eye. If both eyes are eligible, the right eye is included.
- For objective 1: Presence of diabetes will be based on self-reported history or recent medical reports, and a best-corrected visual acuity (BCVA) of 20/30 or better in at least one eye. The cut-off used to classify a participant as diabetic was based on the following criteria: fasting plasma glucose (FPG) ≥126 mg/dL or HbA1c ≥6.5%, or random plasma glucose ≥200 mg/dL [26].
- For objective 2: Presence of diabetes based on self-reported history or based on recent medical reports and BCVA of 20/40 or better, diagnosed to have early DR or early cataract (less than or equal to Lens Opacities Classification System (LOCS) Grade II) in at least one eye. No prior history or diagnosis of any type of DR other than mild NPDR.
- For objective 3: Presence of diabetes will be confirmed either through self-reported history or recent medical records, with a BCVA of 20/100 or better in at least one eye; Reduced vision is only attributed to DR or LOCS cataract grade II. The presence of any stage DR, including non-proliferative diabetic retinopathy (NPDR) or proliferative diabetic retinopathy (PDR) would be included.

The diagnosis of diabetes and the classification of participants as healthy controls or individuals with diabetes would be confirmed later using HbA1c values obtained from systemic investigations.

Exclusion criteria include any congenital colour vision defects, retinal disease other than NPDR and PDR, age-related macular degeneration, glaucoma, history of any ocular surgery in the past 6 months, any laser or retinal treatments, use of medications affecting colour vision (e.g., ethambutol, hydroxychloroquine) and any systemic condition or media opacities (e.g., LOCS Grade III or worse [27] or any neurological disorders) affecting colour perception.

### Sampling

Participants will be recruited from general outpatient departments at the L V Prasad Eye Institute in Hyderabad and diabetes clinics. A purposive sampling strategy will be used to include people with and without diabetes, as well as those with NPDR and PDR. Participant eligibility will be determined using predefined inclusion and exclusion criteria to ensure homogeneity and minimise confounding factors.

### Sample size

The sample size was determined based on effect sizes and correlation estimates derived from prior literature and pilot data, with a desired statistical power of 90% and a two-sided significance level of 0.05. For Objective 1, assuming a minimum expected correlation coefficient of 0.30 between CAD test scores and glycaemic parameters, a sample size of 97 participants per group (diabetic and non-diabetic) was calculated. Accounting for potential dropouts and to ensure robustness, the total sample size was rounded to 200 participants. For Objective 2, based on pilot data demonstrating a moderate effect size (Cohen’s d = 0.66) between early diabetic retinopathy and control groups, at least 49 participants per group were required for 80% power. To counteract any study participation withdrawals during the study, and improve reliability, the total sample size was increased to 100 participants. For Objective 3, to enable the subgroup analysis across different stages of DR and assuming a correlation coefficient of approximately 0.45 between functional (CAD scores), vascular and structural (OCT/OCTA parameters) measures, a total sample size of 200 participants (approximately 50 per severity level) was calculated. Overall, the proposed sample sizes ensure sufficient statistical power to detect clinically meaningful associations, allow subgroup analyses, and account for potential variability

### Study procedures

After the screening phase, the study procedures will be conducted in two stages: a) before dilation and b) after dilation.

### Study procedures done before dilation

Demographic and medical data collection: A proforma containing participants’ demographic details, such as unique medical record number, age, sex, duration of diabetes, HbA1c, medication history, and any previous history of ocular or systemic disease, will be used. Following this, the Indian Diabetic Risk Score Questionnaire [28] will be administered.

Hardy-Rand-Rittler (HRR) colour vision test: The HRR test will be administered to rule out the undiagnosed congenital colour vision defect. Plates 5 and 6 screen for YB defect and Plates 7–10 screen for RG defects. The participant who fails to identify the symbols in any of plates 5–10 will not be included in the study [29].

CAD Test: Colour Assessment and Diagnosis (CAD) Test is a part of a suite of tests available in Advanced Vision and Optometric Tests (AVOT), developed at City, St. George’s University London (formerly City University, London). This is a display-based colour vision test. The CAD test isolates chromatic signals through dynamic noise and provides information on the type and severity of colour vision. The luminance of the checks varied rapidly over time to eliminate luminance cues and isolate chromatic sensitivity. The background stimulus consisted of a central fixation point and a square field of dynamic luminance checks. The background and target (a coloured square) will subtend a visual angle of 5.89° and 1.43° respectively at 1.4 m. The coloured square moves diagonally in one of the four directions (Fig 2B). The participant must indicate the corner to which the coloured square has landed (upper right, upperleft, lower right and lower left), using the response keypad by selecting one of the four buttons (Fig 2C). We will employ randomly interleaved two-down, one-up adaptive staircases to estimate chromatic discrimination thresholds along eight chromatic directions (instead of the 16 chromatic directions used in the conventional CAD test): four red–green (RG) directions (150°, 170°, 330°, and 350°) and four yellow–blue (YB) directions (60°, 64°, 240°, and 244°). The last 6 reversals are averaged and given as a result in thresholds. The results are in standard-normal CAD units, calculated from 330 normal trichromats. One CAD unit is defined as the median chromatic sensitivity threshold of a normal, healthy trichromat [30]. The higher the CAD values, the poorer is the chromatic sensitivity.

**Fig 2:**
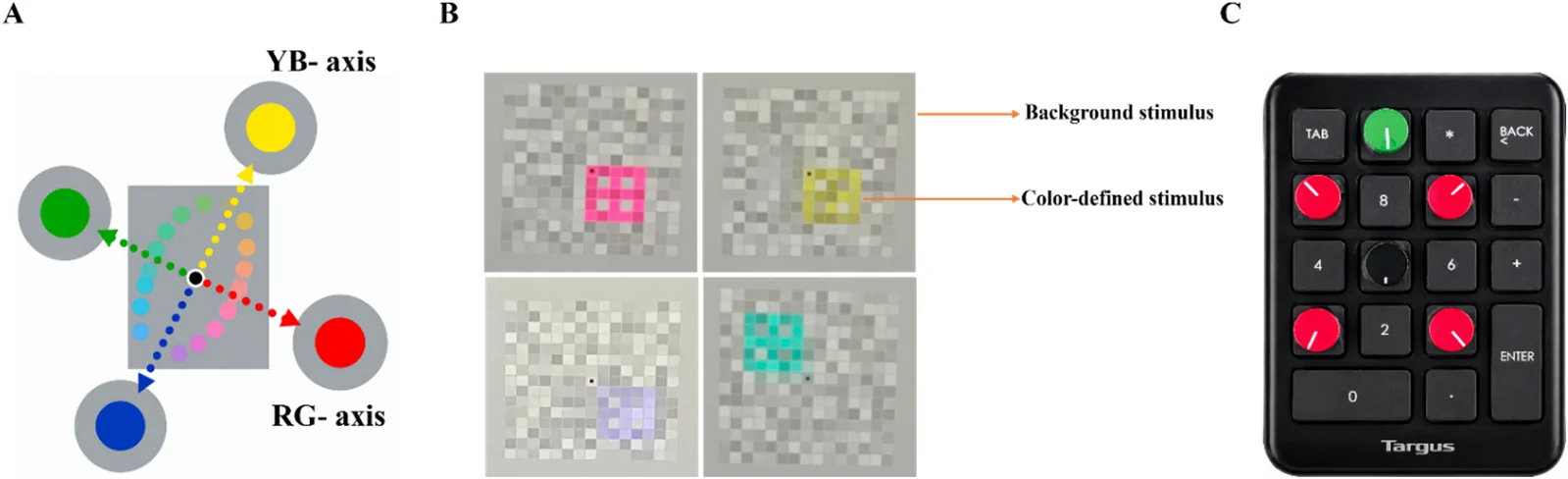
Description of CAD test targets. (A) The yellow-blue and red-green colour isolating axes. (B) Schematic representation of the coloured targets used in the CAD test for assessing red–green (RG) and yellow–blue (YB) color thresholds. (C) The response keypad used by participants to indicate the direction of the target. The response buttons are highlighted in red.

#### Setting up the CAD test.

The setup consists of a laptop operated by the examiner, a calibrated monitor on which the visual stimuli are presented to the participant, and a keypad with buttons mirrored to the stimulus locations. A black curtain separated the examiner and participant to ensure masking during the assessment, and no external light falls on the display. The participant will be seated at a viewing distance of 1.4 meters from the monitor. A monocular CAD test is done with the spectacle correction, if necessary. All the participants would be familiarised with the test procedure using the ‘learning mode’. Participants who complete the ‘learning mode’ without any errors will only be included in the study. Participants who might not be comfortable or have difficulty with the keypad would be assisted by the examiner by pressing the buttons to mimic the verbal responses given by the participant. CAD test would be assessed twice on the same day to measure the intersession test repeatability. RG and YB colour thresholds would be measured, and the average values will be considered for the analysis.

### Study procedures done after dilation

Following the pre-dilatation tests (Fig 3), pupillary dilatation will be achieved using 1% topical tropicamide eye drops (tropicamide 1% w/v) ophthalmic solution, Sunways India Pvt. Ltd., India).

**Fig 3:**
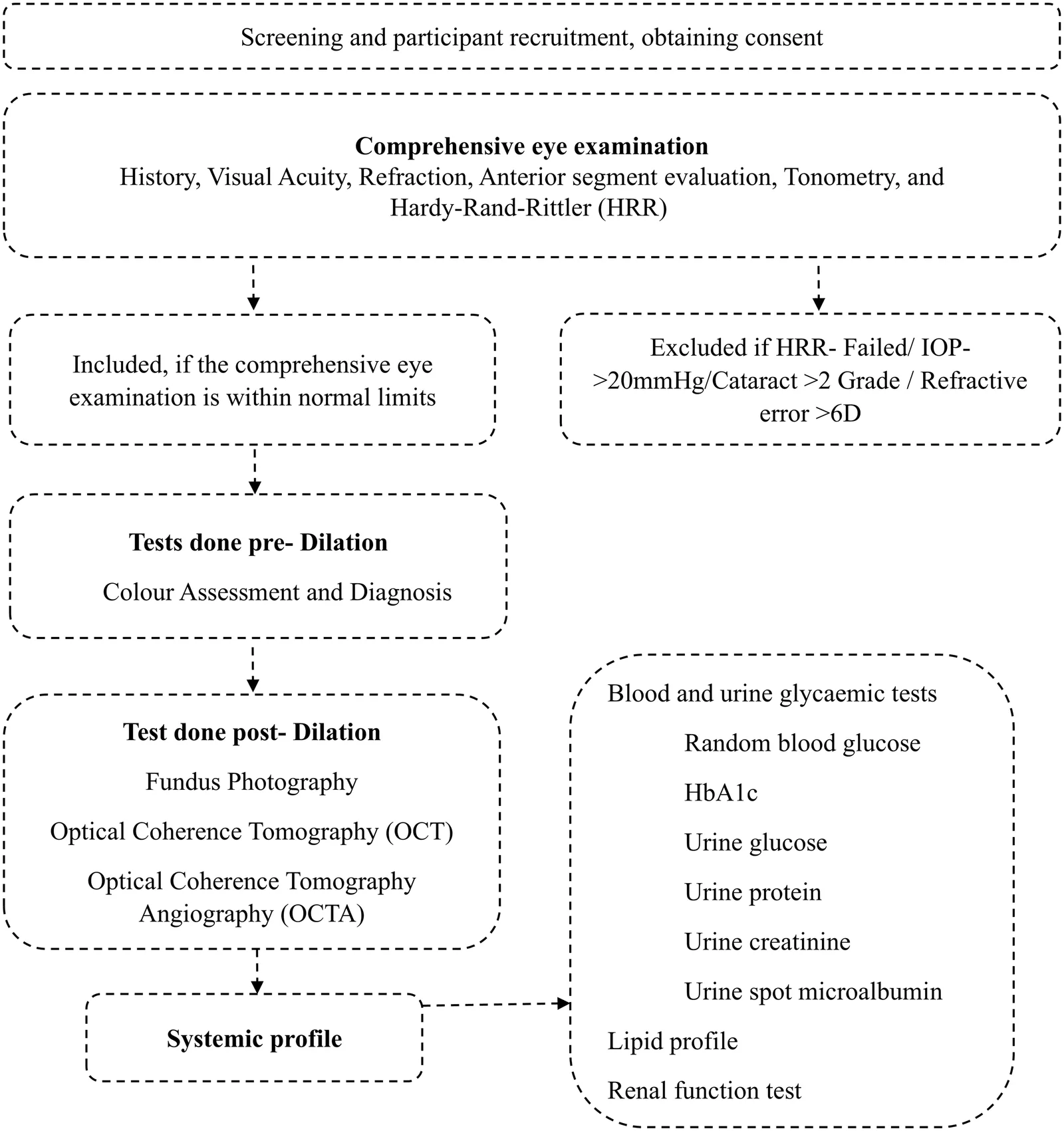
Study workflow showing participant screening, eligibility assessment, colour vision testing, retinal imaging (fundus photography, OCT, and OCTA), and systemic investigations performed for enrolled participants.

Dilated fundus photography: A dilated fundus examination will be performed by a retina specialist. Standard fundus photography will be performed using the Zeiss CLARUS™ 700 (Carl Zeiss Meditech AG, Germany), and DR-related changes will be graded by two certified primary graders who are masked to the final findings. The fundus photographs will then be classified using the International Classification of Diabetic Retinopathy (ICDR) [31]. In cases of disagreement between the grading results, a third masked grader will review the images and provide the final decision.

Dilated slit lamp photography: Dilated slit lamp photography will be performed to grade and document lenticular cataract changes. According to the LOCS-III (27), participants with cataract grades III or higher will be excluded.

Optical Coherence Tomography (OCT) and Angiography (OCTA): OCT will be performed using the Zeiss Cirrus 5000 OCT (Carl Zeiss Meditec, Dublin, CA, USA) to assess subclinical retinal changes, macular thickness, ganglion cell complex (GCC) thickness, and peripapillary retinal nerve fibre layer (RNFL) thickness. Macular scans will be acquired using 512 x 128 protocol over a 6 x 6 mm retinal area centred on the fovea. Peripapillary RNFL thickness and optic nerve head parameters will be obtained using the Optic Disc Cube 200 x 200 scan protocol. Ganglion cell complex analysis will be performed using the Ganglion Cell Analysis (GCA) algorithm derived from the macular cube 512 x 128 protocol. OCTA will be performed to assess the subclinical microvascular changes. Images will be acquired using the 6 × 6 mm angiography scan protocol. OCTA indices with signal strength greater than 7 will be included in the analysis.

Systemic profile: A systemic profile containing blood glucose tests, renal function tests (including blood urea and serum creatinine levels), complete blood count and a lipid profile will be performed.

### Study timeline

The study timeline is provided in Table 2. Following approval from the Institutional Ethics Committee (LEC-BHR-P-12-2025-318) in February 2026, patient recruitment commenced on 1st April, 2026 and will continue until 1st October, 2028. Recruitment and data collection are currently ongoing at the L V Prasad Eye Institute, Hyderabad. Written informed consent will be obtained from all participants prior to the testing. The project is planned over a duration of three years, with one study objective scheduled to be completed each year.

**Table 2:** Proposed three-year study timeline outlining protocol development, data collection for study aims, conference presentation, manuscript preparation, and final report writing.

Year	Year 1		Year 2		Year 3	
	1–6	7–12	13–18	19–24	25–30	31–36
**Pilot testing and protocol development**						
**Aim 1 (data collection)**						
**Conference presentation**						
**Aim 2 (data collection)**						
**Preparation and submission of Manuscript**						
**Aim 3 (data collection)**						
**Report writing and conclusion**						

### Data quality control measures

Ensuring robust quality assurance and control, standardisation will be maintained across all study procedures to ensure data reliability and reproducibility. The CAD test will be conducted under a controlled experimental setting with uniform luminance and testing protocols. All examiners will undergo structured training before commencing the assessment on the study participants. The examiners will be blinded to participants’ glycaemic status and retinal findings, thereby minimising observer bias. Periodic instrument calibration, strict adherence to standard operating procedures, and regular audits of the collected data will be conducted to ensure quality control. Inter- and intra-observer variability will be minimised through standardised testing protocols and consistent application of predefined assessment criteria by trained optometrists.

### Data management

The data will be entered daily using anonymised unique participant codes and stored centrally on a password-protected, online platform to ensure safety. All the parameters will be recorded in a shared online datasheet to ensure updated data and facilitate ongoing monitoring and periodic auditing by the study investigators. Clinical, laboratory, and imaging data will be collected and combined systematically and stored in access-controlled system with access provided only to authorised personnel. Additionally, to minimise transcription errors, routine double data entry and validation checks will be performed. Data will be routinely checked for data ranges, consistency, and outliers before the analysis. Regular data backups will also be maintained to prevent data loss.

### Statistical analysis

D’Agostino-Pearson test will be performed to assess the normality of the data. Descriptive statistics will be used to summarise baseline characteristics, presented as mean ± standard deviation for normally distributed variables and median (interquartile range) for non-normally distributed variables. To determine the association between systemic glycaemic parameters and CAD thresholds, Pearson’s or Spearman’s correlation coefficients will be used, as appropriate. Multiple regression analyses will be performed to assess the independent association between glycaemic parameters and CAD thresholds after adjusting for potential confounders, including age, duration of diabetes and gender.

The diagnostic utility of the CAD test will be assessed by calculating sensitivity, specificity. Receiver Operating Characteristic (ROC) curve analysis will then be used to assess the discriminatory ability of CAD scores for distinguishing participants with DR from those without DR, with the optimal threshold identified using Youden’s index. Cohen’s d will be used to estimate the magnitude of differences in CAD scores across glycaemic categories and to examine differences in relation to OCT- and OCTA-derived structural parameters. Further, the optimal cut-off value will be determined using Youden’s index. Effect size estimation will be performed using Cohen’s d to quantify differences in CAD scores across glycaemic categories, as well as in relation to OCT- and OCTA-derived structural parameters.

### Study outcomes

The study will assess the relationship between CAD test scores and glycemic parameters, including glycated hemoglobin (HbA1c), and random blood glucose levels. The study will assess whether colour vision deficits are associated with glycemic exposure among people with and without diabetes, and explore the potential of CAD as an early functional biomarker for detecting diabetes-related functional changes, even before the onset of clinically evident diabetic retinopathy.

Secondly, the diagnostic performance of the CAD test in detecting early DR will be evaluated using fundus photography as the reference standard. Sensitivity, specificity, and overall diagnostic accuracy will be determined, along with the ability of CAD to discriminate between individuals with early DR and those without diabetic-related retinal changes. Further, the study will look into the relationship between CAD test scores and retinal structural and vascular parameters, including retinal thickness and microvascular changes assessed using OCT and OCTA, respectively, to establish the structure–function relationship in DR.

### Ethics

The proposed study has been commenced after obtaining approval from the Institutional Ethics Committees (IEC) of L V Prasad Eye Institute. Any changes in the protocol will be implemented only after prior approval from the respective Ethics Committees. Written informed consent will be obtained from all participants after providing detailed information regarding all aspects of the study. Participants will be informed that they are allowed to discontinue if at any stage if they are not comfortable before, during, or after data collection. We will also make sure they are aware that the study participation or not, will not have any impact on their clinical care. They can also seek clarification regarding any aspect of the study.

At L V Prasad Eye Institute, written consent is routinely obtained for a comprehensive clinical examination. After the screening stage, once the participants are eligible and agree to participate in the study, a separate consent form will be signed by the participants, ensuring their voluntary participation in the study. Following the tests, all participants’ data will be masked to ensure confidentiality. Exclusive ID codes will be provided. The dataset will not contain any information that is directly related to individual participants. The study data will be safely stored on password-protected computers, with permission to read and retrieve restricted to authorised study investigators. The physical records, such as proformas and signed consent forms, will be maintained in the laboratory’s locked cabinets, with access restricted to the study investigators. Confidentiality of the participant information will be strictly maintained and all the data will be handled securely and anonymised. Data from participants who are excluded from the study will not be included in the analysis.

## Discussion

The current study addresses a critical gap in the early detection of DR by focusing on functional deficits that are known to precede clinically measurable structural changes. Conventional clinical measures, such as visual acuity, lack sensitivity for early retinal changes that spare the macula, highlighting the need for more sensitive biomarkers.

By evaluating the relationship between CAD test scores, glycaemic control, and retinal structural and vascular parameters, this study aims to provide a comprehensive rationale for understanding the early changes associated with retinopathy. The integration of systemic and ocular functional and structural assessments may help improve screening strategies, clinical decision-making and provide an aggregate picture in the management of diabetic retinopathy.

Strengths

- The current study integrates CAD, which provides quantifiable colour vision metric, with robust methodological framework including blinded assessments and adequately powered sample sizes.
- Combining the systemic, functional (CAD), structural (OCT), and vascular (OCTA) parameters provides a holistic understanding of the characteristics and progression of the disease.
- Additionally, the study will be conducted in a high-volume tertiary care setting, ensuring feasibility and clinical relevance.

Limitations

- Since, this is a cross-sectional study, we will not be able to infer any causal relationships.
- Hospital-based recruitment may introduce selection bias. However, strict inclusion and exclusion criteria were designed to reduce bias.

## Conclusion

The current study aims to assess whether the Colour Assessment and Diagnosis (CAD) test can serve as a functional biomarker for the early detection of diabetic retinopathy. Understanding its association with glycaemic exposure and retinal structural and vascular changes remains crucial to incorporate it into routine screening protocols. Early identification of visual functional impairment may help in timely intervention, reduce disease progression, and contribute to the prevention of vision loss in individuals with diabetes.
